# The inpatient burden of abdominal and gynecological adhesiolysis in the US

**DOI:** 10.1186/1471-2482-11-13

**Published:** 2011-06-09

**Authors:** Vanja Sikirica, Bela Bapat, Sean D Candrilli, Keith L Davis, Malcolm Wilson, Alan Johns

**Affiliations:** 1 Shire Pharmaceuticals, Wayne, PA 19087 USA; 2 RTI Health Solutions, 200 Park Offices, Research Triangle Park, NC 27709 USA; 3 The Christie NHS Foundation Trust, Manchester, M20 4BX, UK; 4 Texas Health Care, Fort Worth, TX 76109 USA

**Keywords:** Adhesions, adhesiolysis, abdominal, gynecological, burden of illness, hospitalizations

## Abstract

**Background:**

Adhesions are fibrous bands of scar tissue, often a result of surgery, that form between internal organs and tissues, joining them together abnormally. Postoperative adhesions frequently occur following abdominal surgery, and are associated with a large economic burden. This study examines the inpatient burden of adhesiolysis in the United States (i.e., number and rate of events, cost, length of stay [LOS]).

**Methods:**

Hospital discharge data for patients with primary and secondary adhesiolysis were analyzed using the 2005 Healthcare Cost and Utilization Project's Nationwide Inpatient Sample. Procedures were aggregated by body system.

**Results:**

We identified 351,777 adhesiolysis-related hospitalizations: 23.2% for primary and 76.8% for secondary adhesiolysis. The average LOS was 7.8 days for primary adhesiolysis. We found that 967,332 days of care were attributed to adhesiolysis-related procedures, with inpatient expenditures totaling $2.3 billion ($1.4 billion for primary adhesiolysis; $926 million for secondary adhesiolysis). Hospitalizations for adhesiolysis increased steadily by age and were higher for women. Of secondary adhesiolysis procedures, 46.3% involved the female reproductive tract, resulting in 57,005 additional days of care and $220 million in attributable costs.

**Conclusions:**

Adhesiolysis remain an important surgical problem in the United States. Hospitalization for this condition leads to high direct surgical costs, which should be of interest to providers and payers.

## Background

Adhesions are fibrous bands of scar tissue, often result of surgery, that form between internal organs and tissues, joining them together abnormally [1]. Postoperative adhesions frequently occur following abdominal surgery and are a leading cause of intestinal obstruction. It has been estimated that more than 90% of patients who undergo abdominal operations will develop postoperative adhesions [2].

The most severe complication of postoperative adhesions is small bowel obstruction (SBO), which has a 10% risk of mortality [3,4]. Recent research has demonstrated that readmission episodes averaged 2.7 per patient for SBO or nonspecific abdominal pain (when adhesions were considered likely). Inpatient readmissions accounted for 87% of episodes; 47% of those required repeat surgery [5]. Additionally, in the large retrospective study Surgical and Clinical Adhesions Research, surgical procedures performed on the bowel or the female reproductive system were associated with an increased chance of adhesion development, termed adhesiolysis [6-8]. Ray and colleagues found that 47% of adhesiolysis-related inpatient hospitalizations were for procedures involving the female reproductive tract [2]. Postoperative adhesiolysis-related SBO occurred in 2.8% of patients undergoing hysterectomy for benign conditions and in 5% of those undergoing radical hysterectomy [4,9].

A number of studies have shown that the economic burden of adhesiolysis is significant [2,5,10]. It was estimated that adhesiolysis procedures resulted in 303,836 hospitalizations, 846,415 days of inpatient care, and nearly $1.3 billion in health care expenditures in the United States (US) in 1994 [2]. This cost has decreased when compared with similar data from 1988,[10] due in part to laparoscopic surgery. Despite the decrease in costs associated with laparoscopic surgery, increased use of such techniques did not lead to a decreased rate of overall hospitalizations [2].

Utilizing more recent data, we estimated the current burden of inpatient treatment of adhesiolysis in the US. This study examined the number and rate of adhesiolysis-related hospitalizations, days of care attributable to adhesiolysis, and length of stay (LOS) for adhesiolysis-related hospitalizations, with primary and secondary procedures considered separately. Additionally, we assessed total inpatient costs attributable to adhesiolysis.

## Methods

### Data Source

Data were taken from the 2005 Healthcare Cost and Utilization Project's (HCUP) Nationwide Inpatient Sample (NIS)[11]. The NIS is the largest all-payer inpatient care database in the US and contains data from approximately 8 million hospital stays in 2005. The database also contains clinical and resource use information, including patient demographics, International Classification of Diseases, 9th Revision, Clinical Modification (ICD-9-CM) diagnosis and procedure codes, diagnosis-related group (DRG) codes, LOS, charges, discharge status, payer source, and hospital-specific characteristics. Using the survey design elements provided with the NIS, data can be weighted to produce nationally representative estimates [12]. All financial information in the NIS database is presented as charges rather than costs. To convert hospital charges to costs, facility-specific cost-to-charge ratios were used. Finally, the medical care component of the Consumer Price Index was applied to inflate all financial data to 2007 US dollars [13].

RTI International's Institutional Review Board determined that this study met all criteria for exemption.

### Study Sample

From the NIS, all hospitalizations containing a DRG code of peritoneal adhesiolysis with or without complications (i.e., DRG 150, 151) were defined as primary adhesiolysis-related hospitalizations. Hospitalizations containing a primary or nonprimary ICD-9-CM procedure code for adhesiolysis, but without DRG 150 or 151, were defined as secondary adhesiolysis-related hospitalizations (Table 1). Hospitalizations related to secondary adhesiolysis were stratified by body system, using the following DRG coding:

(1) Digestive system (i.e., DRG 148, 149, 154, or 468),

(2) Hepatobiliary system (i.e., DRG 197, 493, or 494),

(3) Female reproductive system (i.e., DRG 358, 359, 361, or 365),

(4) Pregnancy with evidence of Cesarean section (i.e., DRG 370, 371, or 378).

**Table 1:** Description of Procedure (ICD-9-CM) Codes Used to Identify Adhesiolysis-Related Surgical Procedures

ICD-9-CM Procedure Code	Brief Description
Nongynecologic	
54.5	Lysis of peritoneal adhesions
54.51	Laparoscopic lysis of peritoneal adhesions
54.59	Other lysis of peritoneal adhesions
56.81	Lysis of intraluminal adhesions of ureter
57.12	Lysis of intraluminal adhesions with incision into bladder
57.41	Transurethral lysis of intraluminal adhesions
58.5	Release of urethral structure
59.01	Ureterolysis with freeing or repositioning of ureter for retroperitoneal fibrosis
59.02	Other lysis of perirenal or periureteral adhesions
59.03	Laparoscopic lysis of perirenal or periureteral adhesions
59.11	Other lysis of perivesical adhesions
59.12	Laparoscopic lysis of perivesical adhesions
68.21	Division of endometrial synechiae
Gynecologic	
65.8	Lysis of adhesions of ovary and fallopian tube
65.81	Laparoscopic lysis of adhesions of ovary and fallopian tube
65.89	Other lysis of adhesions of ovary and fallopian tube
70.13	Lysis of intraluminal adhesions of vagina
71.01	Lysis of vulvar adhesions

ICD-9-CM = International Classification of Diseases, 9th Revision, Clinical Modification.

### Study Measures

Study measures included the number of inpatient hospitalizations involving adhesiolysis, adhesiolysis-related hospitalization rates, days of care, and costs attributable to adhesiolysis.

Hospitalization rates per 100,000 persons were assessed using the US Census Bureau's 2005 total US civilian population projection. The total days of care attributable to adhesiolysis were estimated using methods presented by Ray and colleagues that then were adapted for the HCUP NIS [2]. When DRG 150 or 151 (i.e., primary adhesiolysis) was the primary reason for admission, the attributed LOS was simply the mean LOS for this group. For records without a DRG of 150 or 151, excess days attributed to adhesiolysis were calculated as the difference between the mean LOS for those same procedures with adhesiolysis and those procedures without adhesiolysis within each DRG. The total number of adhesiolysis-related days then was estimated as the product of the attributed LOS for the group and the number of adhesiolysis-related hospitalizations within the group.

This study utilized the methodology from Ray and colleagues to estimate the per-day cost attributable to adhesiolysis [2]. Cost per day was estimated by dividing the total cost of adhesiolysis-related hospitalizations divided by the total number of adhesiolysis-related inpatient days. The total inpatient expenditures attributable to adhesiolysis were estimated by multiplying the estimated cost per day attributable to adhesiolysis by the number of days attributed to adhesiolysis.

Average expenditures for surgeon's services were estimated using the Resource-Based Relative Value Scale (RBRVS). The RBRVS value was estimated for Current Procedural Terminology codes related to adhesiolysis (Table 2) and then multiplied by a fixed conversion factor to determine the average surgeon expenditures for each specific procedure. These figures then were inflated to 2007 dollars using the medical care component of the Consumer Price Index.

**Table 2:** Description of Procedure (CPT) Codes Used to Identify Adhesiolysis-Related Surgical Procedures to Estimate Expenditures for Surgeons' Services^a^

CPT Code	Brief Description
44005	Enterolysis (freeing of intestinal adhesion)
50715	Ureterolysis, with or without repositioning of ureter for retroperitoneal fibrosis
50722	Ureterolysis for ovarian vein syndrome
50725	Ureterolysis for retrocaval ureter, with reanastomosis of upper urinary tract or vena cava
58660	Laparoscopy, surgical; with lysis of adhesions (salpingolysis, ovariolysis) (separate)
58559	Hysteroscopy with lysis of intrauterine adhesions (any method)
56441	Lysis of labial adhesions
58740	Lysis of adhesions (salpingolysis, ovariolysis)

CPT = Current Procedural Terminology.

^a ^CPT codes 56304 and 58985 were replaced by code 58660, and CPT code 57451 was retired.

Total inpatient costs attributable to adhesiolysis consisted of inpatient costs and costs for the surgeon's services. Estimates were made separately for primary and secondary adhesiolysis. These also were examined by body system and then aggregated to estimate a total cost. Additionally, inpatient expenditures were summarized to compare Cesarean section deliveries with and without adhesiolysis.

### Statistical Analyses

Descriptive analyses were conducted to display the mean, standard deviation, median, and range of continuous variables, as well as the frequency distribution of categorical variables. All data management and analyses were conducted with SAS and SUDAAN statistical software packages [14,15].

## Results and Discussion

Table 3 illustrates that there were 351,777 adhesiolysis-related hospitalizations in the US in 2005, representing 119 adhesiolysis hospitalizations per 100,000 persons. There were 898 adhesiolysis hospitalizations per 100,000 hospitalizations and 3,549 per 100,000 surgical hospitalizations of any kind (3.5%). Primary adhesiolysis (i.e., DRG 150 or 151) was found in 23.2% of these hospitalizations, while the remaining 76.8% were classified as secondary adhesiolysis (i.e., evidence of the procedure but with a DRG other than 150 or 151).

**Table 3:** Rate of Adhesiolysis-Related Hospitalizations

Characteristic	Estimated Hospitalizations	Rate of Hospitalizations per 100,000 in the US Population^a^	Rate of Hospitalizations per 100,000 Hospitalized Persons^b^	Rate of Hospitalizations per 100,000 Hospitalized Persons for Surgical Intervention^c^
Total number	351,777	118.64	898.22	3,549.04
Adhesiolysis, primary procedure	81,532	27.50	208.18	822.57
Adhesiolysis, secondary procedure	270,245	91.14	690.04	2,726.47

US = United States.

^a ^Based upon the US Census Bureau's 2005 population estimate.

^b ^Among all hospitalizations.

^c ^Among all hospitalized surgical patients.

Table 4 presents background characteristics for the study sample. For primary adhesiolysis, the number of hospitalizations increased steadily by age; for secondary adhesiolysis, the number increased for most age categories. The lowest rate was in patients who were younger than 25 years (5.2 per 100,000 persons for primary adhesiolysis; 13.8 per 100,000 persons for secondary adhesiolysis), and the highest rate was in patients who were older than 65 years (88.4 per 100,000 persons for primary adhesiolysis; 176.7 per 100,000 persons for secondary adhesiolysis). Women had a higher hospitalization rate than men (34.9 vs. 19.7 per 100,000 persons for primary adhesiolysis; 153.1 vs. 13.4 per 100,000 persons for secondary adhesiolysis). Among primary adhesiolysis hospitalizations, almost half (48%) of the patients were admitted via the emergency department, whereas only 20.5% of the secondary adhesiolysis hospitalizations were via the emergency department. Primary adhesiolysis-related hospitalizations were evenly distributed between private insurance and governmental coverage, i.e., Medicaid and Medicare (44% and 48%, respectively), whereas more than half (56%) of the patients with secondary adhesiolysis hospitalizations had private insurance and 37.4% had government-sponsored health care coverage.

**Table 4:** Demographics and Other Patient- and Facility-Specific Characteristics of Interest Among Adhesiolysis-Related Hospitalizations (i.e., DRG 150 or 151) in the US in 2005

	Primary Procedure (N = 81,532)				Secondary Procedure (N = 270,245)			
	
Characteristic	Estimated Hospitalizations	Hospitalizations per 100,000 Population	Rate of Hospitalization (All Hospitalizations)	Rate of Hospitalization (Surgical Hospitalizations)	Estimated Hospitalizations	Hospitalizations per 100,000 Persons	Rate of Hospitalization per 100,000 Hospitalizations	Rate of Hospitalization per 100,000 Hospitalizations With Surgical Procedure
Age (years)								
< 25	5,297	5.15	56.19	456.49	14,212	13.82	150.75	1,224.79
25-34	5,402	13.46	133.39	419.16	46,483	115.79	1,147.76	3,606.77
35-44	11,106	25.32	308.40	888.83	71,062	162.00	1,973.28	5,687.19
45-54	15,691	36.93	372.13	1,142.65	52,732	124.11	1,250.61	3,840.06
55-64	14,324	47.19	322.36	970.96	27,644	91.07	622.13	1,873.86
65-74	13,615	73.00	276.54	877.54	25,980	139.30	527.70	1,674.52
≥ 75	16,034	88.40	189.72	893.19	32,047	176.69	379.20	1,785.20
Missing	64	---	125.96	352.54	86	---	169.25	473.72
Gender								
Female	52,579	34.93	228.80	874.57	230,422	153.07	1002.71	3,832.69
Male	28,696	19.66	178.76	746.39	39,614	13.36	246.77	1,030.37
Missing	256	---	195.29	463.40	208	---	158.68	376.51
Race/ethnicity								
Caucasian	47,344	19.90	241.10	889.04	134,079	56.36	682.79	2,517.78
African-American	6,325	16.69	186.29	920.21	31,153	82.19	917.53	4,532.39
Other^a^	7,398	35.71	133.96	591.38	37,206	179.59	673.71	2,974.15
Missing	20,466	---	192.91	772.80	67,808	---	639.14	2,560.44
Admission source								
ER	38,748	---	232.79	1,553.63	55,369	---	332.64	2,220.06
Another facility	2,274	---	119.81	524.63	4,666	---	245.83	1,076.48
Other^b^	40,509	---	196.45	579.99	210,210	---	1,019.41	3,009.70
Discharge status								
Routine	63,979	---	220.83	865.62	225,752	---	779.22	3,054.37
Transfer to short-term hospital	579	---	68.15	882.96	1,324	---	155.85	2,019.06
Skilled nursing facility	7,252	---	152.54	567.78	16,752	---	352.36	1,311.57
Died in hospital	1,439	---	175.74	989.64	4,662	---	569.34	3,206.17
Other^c^	8,282	---	219.70	802.10	21,755	---	577.11	2,106.94
Primary source of payment								
Medicare	32,085	---	220.41	913.00	63,421	---	435.68	1,804.68
Medicaid	7,445	---	97.42	560.04	37,547	---	491.32	2,824.44
Private Insurance	36,057	---	263.49	848.19	150,852	---	1,102.37	3,548.56
Other^d^	5,853	---	181.10	731.63	18,280	---	565.60	2,285.01
Missing	91	---	186.24	528.76	145	---	296.76	842.53
Hospital region								
Northeast	16,376	29.95	211.20	857.55	55,070	100.71	710.24	2,883.80
Midwest	18,994	28.81	210.56	838.51	57,623	87.39	638.80	2,543.83
South	31,772	29.54	212.63	849.84	108,511	100.89	726.20	2,902.44
West	14,389	21.06	193.21	720.01	49,041	71.76	658.51	2,453.96
Hospital location/teaching status								
Urban	70,728	---	208.10	787.13	237,845	---	699.81	2,646.96
Rural	10,804	---	208.70	1,166.35	32,399	---	625.86	3,497.65
Hospital bed size^e^								
Small	10,532	---	218.05	1,001.92	32,559	---	674.10	3,097.36
Medium	20,062	---	206.81	861.57	63,964	---	659.39	2,746.96
Large	50,938	---	206.78	779.80	173,721	---	705.23	2,659.47
Hospital teaching status								
Teaching	32,737	---	200.09	698.61	108,747	---	664.68	2,320.66
Nonteaching	48,795	---	213.99	933.72	161,498	---	708.23	3,090.37
Hospital control								
Government or private, collapsed	47,163	---	207.20	775.62	155,489	---	683.12	2,557.11
Government, nonfederal, public	5,150	---	197.77	978.84	16,048	---	616.29	3,050.19
Private, nonprofit, voluntary	16,957	---	208.70	832.92	58,180	---	716.06	2,857.76
Private, investor owned	8,419	---	205.72	867.45	30,627	---	748.36	3,155.64
Private, collapsed	3,843	---	243.13	1,286.57	9,901	---	626.39	3,314.69

DRG = diagnosis-related group; HCUP = Healthcare Cost and Utilization Project; NHDS = National Hospital Discharge Survey; US = United States.

^a ^Other category includes Hispanic, Asian/Pacific Islander, Native American, and "other" HCUP category (no further information provided in the data dictionary).

^b ^Other category includes court and law enforcement, and routine, including "other" HCUP category (no further information provided in the data dictionary).

^c ^Other category includes home health, against medical advice, and alive but destination unknown.

^d ^Other category includes self-pay, no charge, and "other" HCUP category (no further information provided in the data dictionary).

^e ^Hospital bed size is based upon facility-specific geographic location and teaching status. These allocations are from the NHDS classification grid.

A total of 967,332 inpatient days of care were attributed to primary and secondary adhesiolysis (Table 5). There were 81,532 hospitalizations and an average LOS of 7.8 days per stay, totaling 632,688 inpatient days of care for primary adhesiolysis. An estimated 334,644 days of care were attributed to secondary adhesiolysis. For hospitalizations in which adhesiolysis was a secondary procedure, we compared the LOS between adhesiolysis and nonadhesiolysis procedures to estimate the LOS attributable to adhesiolysis by each DRG. The majority of DRGs showed an increase in LOS for adhesiolysis hospitalizations versus nonadhesiolysis hospitalizations. On average, hospitalizations related to secondary adhesiolysis resulted in an additional 1.24 hospitalized days compared with nonadhesiolysis-related hospitalizations.

**Table 5:** Inpatient Care Attributable to Abdominal Adhesiolysis by Surgical Procedure in the US in 2005

Reason for Hospitalization	Mean Length of Stay (Days)					
				
(Diagnosis-Related Group)	Adhesiolysis	Nonadhesiolysis	AttributedLOS (Days)	Number of Adhesiolysis-Related Hospitalizations	Attributed Days of Care	Rate of Days Due to Adhesiolysis
Adhesiolysis only (DRG 150, 151)	7.76	---	7.76	81,532	632,688	7.76

**Adhesiolysis as a Secondary Procedure**						

**Digestive System**						

DRG 148: Major small and large bowel procedures with CC	13.87	10.57	3.30	64,588	213,140	3.30
DRG 149: Major small and large bowel procedures without CC	6.30	5.20	1.10	9,313	10,244	1.10
DRG 154: Stomach, esophageal, and duodenal procedures with CC	16.41	11.84	4.57	7,183	32,826	4.57
DRG 468: Extensive OR procedures unrelated to principal diagnosis	16.12	11.25	4.87	3,491	17,001	4.87

Digestive System Total	---	---	---	84,575	273,212	3.23

**Hepatobiliary System**						
DRG 197: Total cholecystectomy without CDE with CC	8.66	8.10	0.56	4,698	2,631	0.56
DRG 493: Laparoscopic cholecystectomy without CDE with CC	5.99	5.21	0.78	9,568	7,463	0.78
DRG 494: Laparoscopic cholecystectomy without CDE without CC	2.70	2.46	0.24	6,811	1,635	0.24

Hepatobiliary System Total	---	---	---	21,077	11,729	0.56

**Female Reproductive System**						

DRG 358: Uterine and adnexa procedures for nonmalignancy with CC	3.90	3.00	0.90	38,263	34,437	0.90
DRG 359: Uterine and adnexa procedures for nonmalignancy without CC	2.46	2.14	0.32	81,543	26,094	0.32
DRG 361: Laparoscopy and incisional tubal interruption	2.80	2.58	0.22	484	106	0.22
DRG 365: Other female reproductive system OR procedures	4.81	5.57	-0.76	4,779	-3,632	-0.76

Female Reproductive System Total	---	---	---	125,069	57,005	0.46

**Pregnancy, C-Section**						

DRG 370: Cesarean section with CC	4.30	4.45	-0.15	9,901	-1,485	-0.15
DRG 371: Cesarean section without CC	3.12	3.37	-0.25	26,011	-6,503	-0.25
DRG 378: Ectopic pregnancy	2.16	1.97	0.19	3,612	686	0.19

Pregnancy, C-section Total	---	---	---	39,524	-7,302	-0.18

Total, Adhesiolysis as a secondary procedure	---	---	---	270,245	334,644	1.24
Total, all adhesiolysis-related procedures	---	---	---	351,777	967,332	2.75

CC = complications and comorbidities; DRG = diagnosis-related group; LOS = length of stay; US = United States.

The difference in mean LOS was greatest for extensive operation room procedures unrelated to principal diagnosis (i.e., DRG 468), with 4.9 days attributable to adhesiolysis. For stomach, esophageal, and duodenal procedures with complications of comorbid conditions (i.e., DRG 154), 4.6 days were attributable to adhesiolysis. Almost half (46.3%) of all secondary adhesiolysis procedures (125,069) were female reproductive tract related, resulting in 57,005 days of care. Thus, 0.46 day of additional stay were attributable to adhesiolysis. The longest LOS for female reproductive system procedures was for DRG 358 (uterine and adnexa procedures for nonmalignancy), which resulted in an additional day of inpatient stay (0.90 day).

Table 6 shows that total inpatient expenditures for adhesiolysis-related hospitalizations were $2.25 billion: of this amount, primary adhesiolysis-related hospitalizations accounted for $1.35 billion and secondary adhesiolysis-related hospitalizations accounted for $902 million. Of the total secondary adhesiolysis expenditures, $622 million (69%) were related to procedures for the digestive system and $220 million (24.3%) were related to procedures for the female reproductive system. Adhesiolysis related to the hepatobiliary system and pancreas and Cesarean sections accounted for $41 million and $18 million, respectively.

**Table 6:** Inpatient Expenditures Attributable to Abdominal Adhesiolysis in the US in 2005

Expenditure	Attributed to Adhesiolysis	Total in Millions (2007 $)
By type of procedure		
Adhesiolysis as primary procedure		
Total days of care	632,688	$1,277
Surgical procedures	81,532	$68
Subtotal	---	$1,345
Adhesiolysis as secondary procedure		
Total days of care	334,644	$675
Surgical procedures	270,245	$227
Subtotal	---	$902
Cost stratification of secondary adhesiolysis, by body system		
Digestive system		
Total days of care	273,212	$551
Surgical procedures	84,575	$71
Subtotal	---	$622
Hepatobiliary system and pancreas		
Total days of care	11,729	$24
Surgical procedures	21,077	$18
Subtotal	---	$41
Female reproductive system		
Total days of care	57,005	$115
Surgical procedures	125,069	$105
Subtotal	---	$220
Pregnancy, C-sections		
Total days of care	-7,302	-$15
Surgical procedures	39,524	$33
Subtotal	---	$18

Total expenditures	---	$2,247

US = United States.

The rate of adhesiolysis-related hospitalizations in the US has remained fairly constant from 1998 to 2005: from 115.5 in 1988 [10] to 117.3 in 1994 [2] and ultimately 118.6 per 100,000 persons in 2005. In these same time periods, the average LOS for primary adhesiolysis-related hospitalizations has steadily decreased from 11.2 days to 9.7 days and 7.8 days, respectively. The costs for such hospitalizations, when inflated to reflect 2007 dollars, indicated an increase of $112 million between 1988 and 2005, despite the 3.4-day (or 30%) decrease in LOS--this represented a 5% increase in medical care costs. This increase suggested that costs of treating adhesiolysis have increased substantially.

Primary adhesiolysis contributed 23% of all adhesiolysis procedures (81,532) but represented more than half of the total cost burden ($1.3 billion). Secondary adhesiolysis was substantially higher in volume, representing 77% of procedures (270,245) but less half of the total cost burden ($902 million). The greatest number of procedures was to the female reproductive tract (125,069) while procedures to the digestive tract yielded the highest overall costs ($622 million).

Potentially mitigating this growth in the cost of adhesiolysis may be the continuing trend in the US toward minimally invasive and laparoscopic approaches, which may lessen the occurrence of postoperative adhesions [2]. Although laparoscopy reduces surgical trauma, the procedure has not been show to reduce the incidence of adhesion-related readmissions [16].

This study is subject to potential limitations consistent with retrospective database studies. Conditions and events of interest were identified by diagnosis codes. Previous research has suggested that the condition may be underreported [17]. This may mean that the actual cost of adhesiolysis-related disease is greater than the estimate provided by our study. The database used for this study was not specifically designed to assess inpatient burden. Like all administrative billing databases, the data contained in the HCUP NIS are dependent upon the quality of coding, which may be influenced by reimbursement incentives. However, we do not feel it likely that such incentives greatly affected our results since the majority of overall adhesiolysis costs were a part of secondary adhesiolysis procedures and not the more costly primary adhesiolysis. Moreover, even if such incentives exist and are reflected in the data used for this study, these data are indicative of real world practice. Additionally, with such a large sample, the effect of any coding errors or anomalies would likely be minimized.

Furthermore, due to the nature of the database, detailed clinical characteristics could not be ascertained; therefore, the results could not be adjusted for disease severity or other clinical parameters. However, it is unlikely that these factors would have had a large impact on the results, as this study focused on those patients receiving inpatient care. Additionally, since the database contains US data only, the results may not be generalizable to other populations outside of the US. Lastly, because the focus of this study was on direct cost measures, the results do not account for productivity loss for the patient or caregiver and potential future societal contributions that may be lost due to death resulting from or related to adhesiolysis. Because we examined only the direct health care costs associated with inpatient adhesiolysis, we have not examined any adhesiolysis-related surgeries performed at other sites of care, such as ambulatory surgical centers. Further, our study does not capture direct costs relating to but occurring before or after surgery, including pain medications, cost of work-up visits, and procedures related to diagnosis. Similarly, patient work-ups and diagnostic laparoscopic procedures that may have occurred at separate visits and prior to the adhesiolysis surgery were not captured if specific DRG codes were not listed for those hospitalizations [6,7,9]. Hence, this study's estimates of costs are likely to be conservative.

## Conclusions

Adhesions remain an important surgical problem, and hospitalization for adhesiolysis leads to a high direct cost burden in the US. Despite a trend of decreasing LOS for adhesiolysis-related hospitalizations from 2001 to 2005, adhesiolysis-related costs continue to rise even while the overall rate of adhesiolysis procedures remains constant. Consistent with previous research, the distribution of inpatient care and costs across the diagnostic categories remained steady from 2001 to 2005, with only a slight increase in primary adhesiolysis procedures over time. From 2001 to 2005, hospitalizations for adhesiolysis related to the digestive system and to the female reproductive tract had the largest number of inpatient days and accounted for the majority of costs related to secondary adhesiolysis procedures.

Adhesiolysis remains a substantial economic burden to the US health care system, which should be of interest to providers and commercial and government payers. Further research incorporating detailed clinical data and indirect costs would aid in a greater understanding of the overall burden of adhesiolysis.

## Competing interests

VS was an employee of Ethicon, Inc. at the time that this manuscript was prepared; he is currently an employee of Shire Pharmaceuticals. BB, SDC, and KLD are employees of RTI Health Solutions, the research organization contracted by Ethicon to conduct this study. AJ is an employee of Texas Healthcare; MW is an employee of Christie NHS Foundation Trust.

## Authors' contributions

VS was responsible for developing the study design, interpreting the analysis results, and drafting the manuscript text; he is the primary author of this manuscript. BB, SDC, and KLD were responsible for the acquisition, management, interpretation, and analysis of all study data. BB, SDC, and KLD also assisted with developing the study design, interpreting the analysis results, and drafting the manuscript. AJ and MW contributed clinical expertise and guidance and assisted in interpreting the analysis results and drafting the manuscript text.

All authors confirm that they have read the journal's position on issues involved in ethical publication and affirm that this research report is consistent with those guidelines. Finally, all authors have read and approved the final manuscript.

## Funding

This study and the preparation of this manuscript were funded by Ethicon, Inc. The authors acknowledge that Ethicon, Inc. is the maker of GYNECARE INTERCEED, a product that is marketed to prevent pelvic adhesions.

## Pre-publication history

The pre-publication history for this paper can be accessed here:

http://www.biomedcentral.com/1471-2482/11/13/prepub

## References

[B1] Beck DE Understanding abdominal adhesions Ostomy Q 2001 38 2 50 51

[B2] Ray NF Denton WG Thamer M Henderson SC Perry S Abdominal adhesiolysis: inpatient care and expenditures in the United States in 1994 J Am Coll Surg 1998 186 1 1 9 10.1016/S1072-7515(97)00127-0 9449594

[B3] Menzies D Parker M Hoare R Knight A Small bowel obstruction due to postoperative adhesions: treatment patterns and associated costs in 110 hospital admissions Ann R Coll Surg Engl 2001 83 40 46 11212449 PMC2503561

[B4] diZerega GS Tulandi T Prevention of intra-abdominal adhesions in gynaecological surgery Reprod Biomed Online 2008 17 303 306 10.1016/S1472-6483(10)60211-8 18764998

[B5] Tingstedt B Isaksson J Andersson R Long-term follow-up and costs analysis following surgery for small bowel obstruction caused by intra-abdominal adhesions Br J Surg 2007 94 743 748 10.1002/bjs.5634 17330836

[B6] Ellis H Moran BJ Thompson JN Parker MC Wilson MS Menzies D McGuire A Lower AM Hawthorn RJ O'Brien F Buchan S Crowe AM Adhesion-related hospital readmissions after abdominal and pelvic surgery: a retrospective cohort study Lancet 1999 353 1476 1480 10.1016/S0140-6736(98)09337-4 10232313

[B7] Parker MC Ellis H Moran BJ Thompson JN Wilson MS Menzies D McGuire A Lower AM Hawthorn RJ O'Briena F Buchan S Crowe AM Postoperative adhesions: ten-year follow-up of 12,584 patients undergoing lower abdominal surgery Dis Colon Rectum 2001 44 822 829 10.1007/BF02234701 11391142

[B8] Lower AM Hawthorn RJ Ellis H O'Brien F Buchan S Crowe AM The impact of adhesions on hospital readmissions over ten years after 8489 open gynaecological operations: an assessment from the Surgical and Clinical Adhesions Research Study Br J Obstet Gynaecol 2000 107 855 862 10.1111/j.1471-0528.2000.tb11083.x 10901556

[B9] Meagher AP Moller C Hoffmann DC Non-operative treatment of small bowel obstruction following appendectomy or operation on the ovary or tube Br J Surg 1993 80 1310 1311 10.1002/bjs.1800801030 8242308

[B10] Ray NF Larsen JW Stillman RJ Jacobs RJ Economic impact of hospitalizations for lower abdominal adhesiolysis in the United States in 1988 Surg Gynecol Obstet 1993 176 271 276 8438200

[B11] HCUP Nationwide Inpatient Sample. Healthcare Cost and Utilization Project (HCUP) http://www.hcup-us.ahrq.gov/nisoverview.jsp

[B12] Steiner C Elixhauser A Schnaier J The Healthcare Cost and Utilization Project: an overview Eff Clin Pract 2002 5 3 143 151 12088294

[B13] US Bureau of Labor Statistics. Consumer Price Index for medical services http://data.bls.gov/PDQ/outside.jsp?survey=cu

[B14] SAS Institute Inc SAS 9.1.3 2003 Cary, NC: SAS Institute Inc

[B15] Research Triangle Institute SUDAAN (Release 9.0.1) 2005 Research Triangle Park, NC: Research Triangle Institute

[B16] Gutt CN Oniu T Schemmer P Mehrabi A Büchler MW Fewer adhesions induced by laparoscopic surgery? Surg Endosc 2004 18 6 898 906 10.1007/s00464-003-9233-3 15108105

[B17] Parker MC Wilson MS Menzies D Sunderland G Clark DN Knight AD Crowe AM Surgical and Clinical Adhesions Research (SCAR) Group The SCAR-3 study: 5-year adhesion-related readmission risk following lower abdominal surgical procedures Colorectal Dis 2005 7 551 558 10.1111/j.1463-1318.2005.00857.x 16232234

